# An esophageal stent integrated with wireless battery-free movable photodynamic-therapy unit for targeted tumor treatment

**DOI:** 10.1016/j.mtbio.2024.101394

**Published:** 2024-12-09

**Authors:** Qian Han, Pingjin Zou, Xianhao Wei, Junyang Chen, Xiaojiao Li, Li Quan, Ranlin Wang, Lili Xing, Xinyu Xue, Yi Zhou, Meihua Chen

**Affiliations:** a School of Physics, University of Electronic Science and Technology of China, Chengdu, 611731, China; b Department of Radiation Oncology, Radiation Oncology Key Laboratory of Sichuan Province, Sichuan Clinical Research Center for Cancer, Sichuan Cancer Hospital & Institute, Sichuan Cancer Center, Affiliated Cancer Hospital of University of Electronic Science and Technology of China, Chengdu, 610041, China; c School of Medicine, University of Electronic Science and Technology of China, Chengdu, 610054, China; d Chengdu University of Traditional Chinese Medicine, Chengdu, 610032, China; e Department of Endoscopy, Sichuan Clinical Research Center for Cancer, Sichuan Cancer Hospital & Institute, Sichuan Cancer Center, Affiliated Cancer Hospital of University of Electronic Science and Technology of China, Chengdu, 610041, China; f Department of Abdominal Oncology, Sichuan Clinical Research Center for Cancer, Sichuan Cancer Hospital & Institute, Sichuan Cancer Center, Affiliated Cancer Hospital of University of Electronic Science and Technology of China, Chengdu, 610041, China

**Keywords:** Cancer treatment, Electrochemical pneumatic soft actuator, Battery-free, Wireless control, Photodynamic therapy

## Abstract

Esophageal cancer is the eighth most common cancer worldwide and the sixth leading cause of cancer-related deaths. In this study, we propose a novel esophageal stent equipped with a wireless, battery-free, and movable photodynamic therapy (PDT) unit designed to treat esophageal tumors with flexibility, precision, and real-time control. This system integrates a PDT unit and an electrochemical pneumatic soft actuator into a conventional esophageal stent. Each module incorporates a piezoelectric transducer capable of receiving external ultrasound to power the respective module. These transducers selectively respond to different external ultrasound frequencies, enabling independent operation without mutual interference. The therapy module provides a light source for PDT, inducing the production of cytotoxic reactive oxygen species (ROS) in tumor cells and promoting apoptosis. The pneumatic actuator based on electrochemical principles plays a critical role in controlling the position of the PDT light source, enabling the movement of the therapy module up to 200 mm within 15 min. This allows real-time control to maintain the light source near the tumor, ensuring precise and targeted treatment. The system can wirelessly and in real-time control the PDT light source's position via external ultrasound, offering a novel approach for treating esophageal cancer patients according to the need of tumor's progression.

## Highlights

• Development of an esophageal stent integrated with a photodynamic therapy unit for targeted tumor treatment.
• A miniature implantable, movable wireless μ-LED provides the light source for PDT.
• The electrochemical pneumatic soft actuator enables flexible control of the light source position.
• The tumor cell apoptosis is efficient upon the PDT process.

## Graphical abstract

We propose a novel esophageal stent equipped with a wireless, battery-free, and movable photodynamic therapy (PDT) unit designed to treat esophageal tumors with flexibility, precision, and real-time control. The system can wirelessly and in real-time control the PDT light source's position via external ultrasound, offering a novel approach for treating esophageal cancer patients according to the need of tumor's progression.

**Figure:**
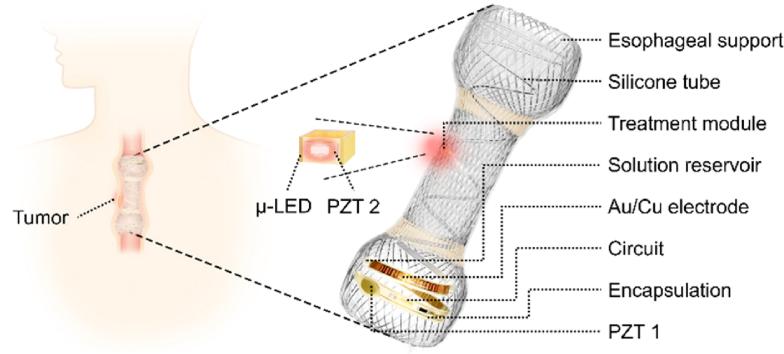

## 1 Introduction

Esophageal cancer is the eighth most common cancer worldwide and the sixth leading cause of cancer-related deaths [1]. Statistics show that the 5-year survival rate is only 15–20 %, leading to over 500,000 deaths annually [2,3]. By 2040, the global incidence of esophageal cancer is expected to reach 987,723 new cases, with 914,304 deaths [4]. Current treatment modalities include surgical resection, radiation therapy, chemotherapy, and palliative care [5,6]. Surgical resection of esophageal tumor tissues usually necessitates removal of the larynx, impairing vocal function and potentially leading to postoperative complications [5,7,8]. Both radiation and chemotherapy have the widespread use, but lack selectivity for tumor cells and can result in severe side effects such as radiation pneumonitis, pleural effusion, and pericardial effusion [[9], [10], [11]]. Consequently, there is an urgent need for novel therapeutic approaches aimed at improving survival rates and quality of life for esophageal cancer patients.

In this context, photodynamic therapy (PDT) has emerged as a promising innovative treatment due to its higher selectivity and fewer systemic adverse effects [[12], [13], [14], [15], [16], [17], [18], [19], [20], [21], [22]]. However, the conventional use of implanted optical fibers for PDT is plagued by complex equipment, cumbersome procedures, and increased patient discomfort, highlighting the need for more portable and effective light delivery systems for the esophageal cancer patients [[23], [24], [25]]. Recently, some research groups have explored small devices for PDT, embedding light-emitting components within the body and powering them through wireless method to achieve effective internal illumination of living tissues [[26], [27], [28], [29], [30], [31], [32], [33], [34]]. For esophageal cancer patients, tumor progression often leads to esophageal stenosis, causing eating difficulties [[35], [36], [37]]. Esophageal stent placement is a widely used minimally invasive intervention that rapidly relieves dysphagia and obstruction symptoms, improving nutritional status and being widely employed in clinical treatments [[38], [39], [40], [41]]. Therefore, the integration of small PDT unit and commonly used esophageal stricture-relieving stent may be a suitable system for the novel internal esophageal tumor treatment [42].

Furthermore, it is also needed to dynamically adjust the position of the PDT unit based on the tumor's status in the esophageal to achieve efficient and precise treatment. The tumors have proliferative and metastatic properties, and during treatment, tumor progression can enlarge the lesion area or cause metastasis [[43], [44], [45], [46], [47], [48], [49], [50]], weakening the therapeutic effect on regions distant from the light source. Soft robotics research has shown significant potential in the medical field, with flexible structures and excellent deformability suitable for operations in unknown and unstructured environments [[51], [52], [53], [54]]. Developing soft actuators is a core task in soft robotics research, primarily responsible for driving or controlling the systems [55,56]. Among different driving mechanisms, pneumatic soft actuator is simple in structure, cost-effective, highly efficient, quick in response, and environmentally friendly [57,58]. Previously, our team used electrochemical pneumatic soft actuator to perform in vivo surgeries, e.g. inducing eye shape changes for the treatment of high myopia [59]. Consequently, the pneumatic soft actuator for moving the PDT unit is suitable to integrate into the system.

Here, we propose a wireless, battery-free, multifunctional therapeutic system that integrates a PDT module and an electrochemical pneumatic soft actuator into an esophageal stricture-relieving stent. This system not only alleviates esophageal stenosis symptoms and rapidly improves swallowing difficulties but also achieves precise and targeted treatment of tumor cells. The system comprises an esophageal stent, two piezoelectric transducers, an electrochemical pneumatic soft actuator, a micro light-emitting diode (μ-LED), flexible circuits, and biocompatible packaging. The μ-LED serves as the treatment module, providing a light source for PDT to activate photosensitizers that produce cytotoxic reactive oxygen species (ROS). The electrochemical pneumatic soft actuator, consisting of an electrolysis chamber and a long soft silicone tube track, houses the treatment module inside the track, allowing unidirectional movement along the track, with the entire actuator spirally wound inside the esophageal stent. When the tumor grows or metastasizes, the actuator can move the μ-LED to the new tumor site. The piezoelectric transducers convert external ultrasound waves into electrical energy, powering the therapeutic and actuation processes. These two processes are independently controlled by two piezoelectric transducers, each selectively responding to different external ultrasound frequencies, allowing independent and non-interfering operation. This innovative therapeutic approach holds promise for providing more effective and personalized treatment options for esophageal cancer patients, offering new avenues and methods for clinical intervention.

## 2 Result and discussion

### 2.1 Structure and operation of the stent system

**Fig. 1:**
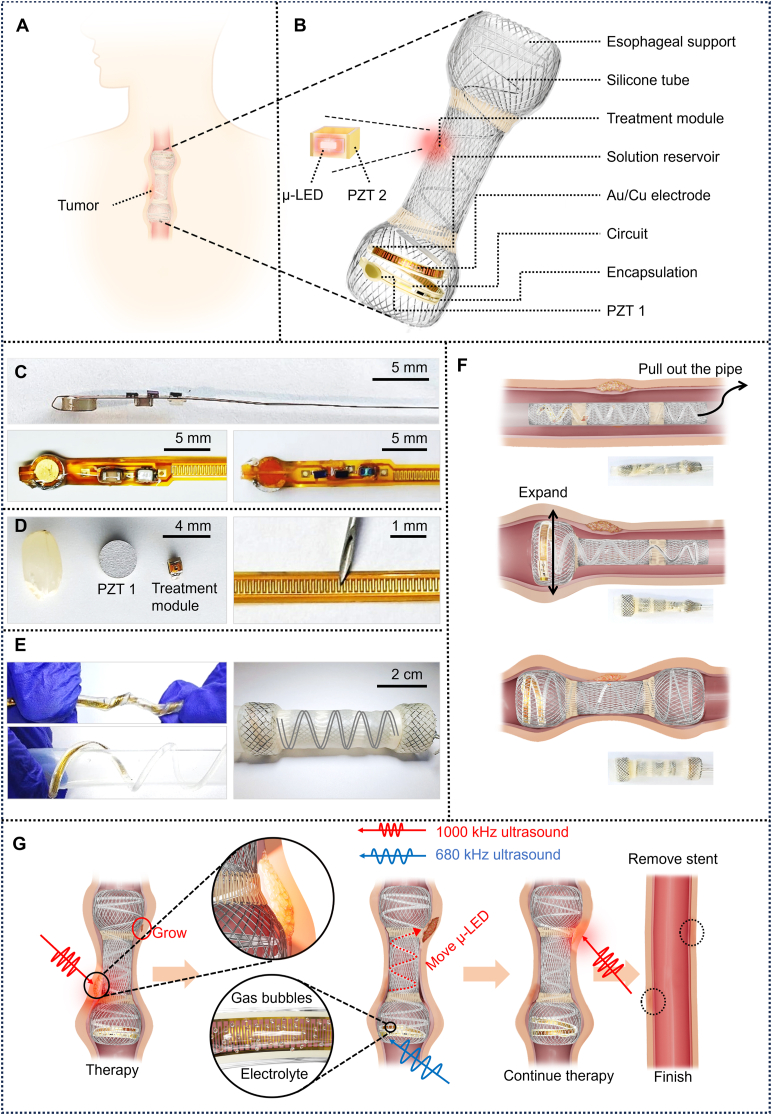
Overview of the esophageal stent for tumor treatment. (A) The stent installed in the affected area. (B) The structure of the stent. (C) Photograph showing various configurations of the actuator. (D) The sizes of PZT 1 and the therapy module, smaller than a grain. The width and gap of the interdigitated electrodes are 80 μm, finer than a syringe needle. (E) The actuator can be twisted and bent, demonstrating its capability to integrate into the esophageal stent. (F) The process of implanting the stent into the cancerous area. (G) The treatment process of the esophageal cancer using the movable PDT stent system.

Fig. 1 illustrates the structure and operation of the wireless, battery-free, and movable PDT system designed for esophageal tumor treatment. The system is installed at the site of esophageal stenosis caused by tumor growth, as shown in Fig. 1A. Fig. 1B depicts the overall structure of the system, which integrates a commercial nitinol esophageal stent as the framework, a therapy module that provides the PDT light source, and an electrochemical pneumatic soft actuator that supplies moving force and guidance for the therapy module. The wireless unit comprises two high-sensitivity lead zirconate titanate (PZT) piezoelectric transducers: PZT 1 and PZT 2. These transducers harvest energy from ultrasound waves and convert it into electrical power for the respective modules. The therapy module, consisting of PZT 2 and a μ-LED, utilizes the energy collected by PZT 2 to illuminate the μ-LED, providing the light source for PDT. The electrochemical pneumatic soft actuator comprises PZT 1, a control circuit, interdigitated electrodes, an electrolysis chamber, ionic solution, a track tube, and a piston. Using the energy harvested by PZT 1, the sophisticated configuration of the interdigitated electrodes and ionic solution induces electrolysis within the sealed chamber, generating bubbles that cause piston displacement, thereby controllably adjusting the position of the therapy module along the actuator track. The actuator is helically wound and adhered to the inside of the esophageal stent using a biocompatible flexible polymer (polydimethylsiloxane, PDMS). The helical structure ensures maximum coverage of the track within the stent, maximizing the area reachable by the therapy module. The entire system weighs 4.8 g and the integrated stent measures 7.8 cm in length. This compact and lightweight design facilitates minimally invasive implantation.

Fig. 1C shows the integrated design of the electrochemical actuator, which includes the control circuit, energy module, and interdigitated electrodes on a flexible circuit board. Fig. 1D demonstrates that the volume of PZT 1 is smaller than a grain of rice, effectively minimizing the patient's sensation of a foreign object. The treatment module's compact size ensures frictionless movement within the actuator track. The interdigitated electrodes, fabricated using microfabrication techniques, have a width and gap of 80 μm, finer than a syringe needle. Fig. 1E highlights the flexibility of the actuator's solution storage chamber, circuit, and track, which can be twisted many times without performance degradation, ensuring the maintenance of the helical structure within the stent.

Fig. 1F illustrates the implantation process of the system into the esophagus of an esophageal cancer patient. The system is folded and compressed into a sufficiently thin catheter, which is then entirely implanted into the cancerous region of the esophagus. Upon removal of the catheter, the stent rapidly expands and props the esophagus.

Fig. 1G outlines the operating procedure of the system. Ultrasound at 1 MHz is directed at PZT 2, activating it and lightning the μ-LED. The 660 nm red light irradiates the tumor area, inducing the production of ROS in the tumor cells, leading to tumor cell apoptosis. After treating this area, 680 kHz ultrasound is directed at PZT 1 to activate the actuator, moving and positioning the therapy module at the next tumor site. The process is repeated, treating each tumor site sequentially until all affected areas are cleared, after which the entire system is removed.

### 2.2 The electrochemical pneumatic soft actuator

**Fig. 2:**
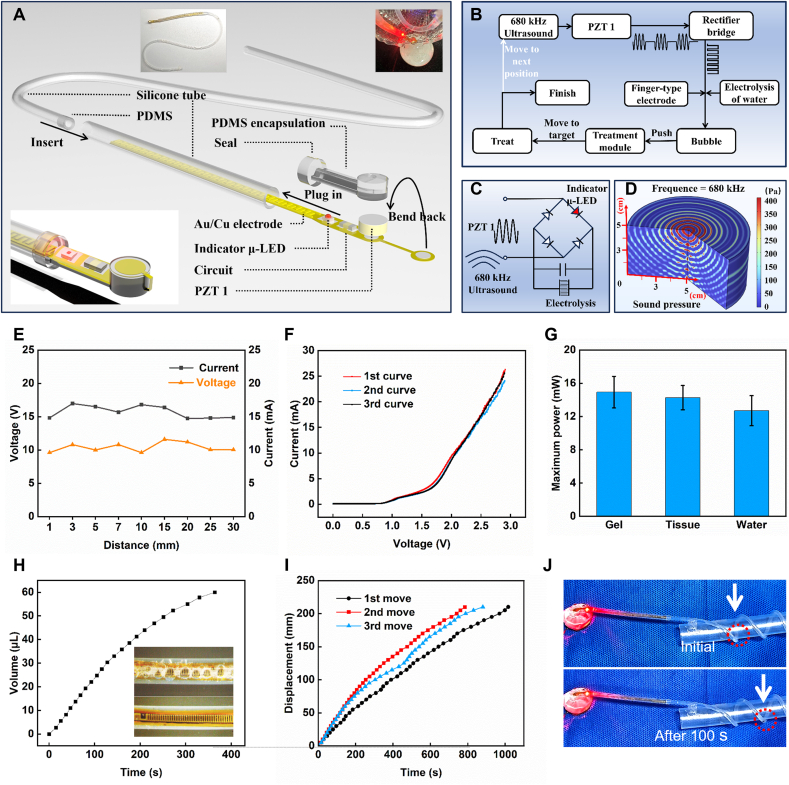
Structure and performance of the electrochemical actuator. (A) Exploded view and fabrication process of the actuator. (B) Workflow of the actuator in treating esophageal cancer. (C) Schematic of the actuator's energy harvesting circuit. (D) Simulation of 680 kHz ultrasound propagation. (E) Short-circuit current and open-circuit voltage peaks of PZT 1 at distances of 1 – 30 mm from the ultrasound source. (F) Current-voltage characteristics of the electrochemical actuator within the voltage range of 0 – 3 V. (G) Output power of the electrochemical actuator in different media after receiving ultrasound. (H) Volume of gas produced by the solution electrolysis. (I) Displacement of the therapy module caused by the actuator's operation. (J) Optical image of the therapy module displacement within 100 s.

Fig. 2 shows the characterization of the electrochemical pneumatic soft actuator. Fig. 2A illustrates the exploded view and assembly process of the actuator. Given the excellent biocompatibility and flexibility of silicone [60,61], tubes of various diameters and lengths were selected to construct the actuator's transparent solution reservoir and control channels. Essential electronic components, including PZT 1, were soldered onto a custom-designed flexible circuit board, which was then encapsulated with PDMS to ensure biocompatibility for in vivo applications [[62], [63], [64]]. PZT 1, a cylindrical component with a diameter of 3 mm and a height of 1 mm, operates at a resonance frequency of 680 kHz.

To enhance electrochemical performance, we opted for interdigitated electrodes with smaller gaps and larger opposing surface areas. This design provides a stronger electric field for the electrolysis reaction: 2H₂O (liquid) → O₂ (gas) + 2H₂ (gas), thereby accelerating gas generation. Simultaneously, the gold electrodes, which exhibit stable chemical properties, ensure stable conditions for the electrolysis process. These interdigitated electrodes are sealed within a solution reservoir, printed on both sides of the circuit, and extend throughout the solution storage channel to guarantee complete immersion in the ionic solution. Following assembly, the ionic solution is injected into the storage tank, and a Vaseline piston is placed within the guide.

To maintain sufficient conductivity, a 50 mmol/L NaOH solution was employed as the electrolyte [65]. Sodium hydroxide (NaOH), a strong base, increases the concentration of OH⁻ ions, providing a higher availability of reactive ions at the electrodes. This facilitates current flow through the electrolyte, thus enhancing the actuator's electrochemical activity.

Fig. 2B shows the actuator's operation. A 680 kHz ultrasound source with a 70 % duty cycle activates PZT 1, which converts the ultrasound waves into electrical energy through the piezoelectric effect. The electrical signal is rectified and regulated before being transmitted to the interdigitated electrodes, inducing electrolysis in the sealed electrolysis chamber's ionic solution. This generates bubbles that push the piston in the track, positioning the therapy module near the tumor for precise treatment. If the tumor grows or metastasizes, reducing treatment effectiveness in distant areas, the actuator can reposition the therapy module to the new target location for efficient treatment. Fig. 2C illustrates the circuit structure, where the rectifier bridge and capacitor provide a stable DC signal for the electrolysis of the ionic solution within the actuator. One Schottky diode in the rectifier bridge is replaced with a μ-LED, which serves as an indicator while maintaining rectification functionality. The dynamic process is shown in Movie S1.

Fig. 2D simulates the sound pressure propagation of 680 kHz ultrasound in water, which has acoustic properties similar to human tissue (Z_water = 1.48 MRayl, Z_tissue = 1.63 MRayl, average for human tissue). The simulation results indicate that ultrasound can easily transmit 5 cm through tissue with minimal attenuation, and the system can be remotely driven and collect stable ultrasound energy during operation. Fig. 2E demonstrates the stable output of PZT 1 at different depths in various media, with the open-circuit voltage peak remaining around 10 V and the short-circuit current peak stabilizing around 15 mA, ensuring the device's reliable performance. The output of PZT 1 with a 70 % ultrasound duty cycle is shown in Fig. S1 (Supporting Information), and the current output with a 100 % duty cycle is shown in Fig. S2 (Supporting Information).

Fig. 2F shows the current-voltage characteristics of the actuator within a voltage range of 0–3 V. The device starts operating at 1.0 V, with the interdigitated electrodes in the actuator promoting the electrolysis reaction. To ensure adequate conductivity, a 50 mmol/L NaOH solution is used as the electrolyte. Fig. 2G presents the actuator's power output at a depth of 10 mm in different media, with a power output of 14.9 mW in gel, 14.2 mW in tissue, and 12.7 mW in water. Fig. 2H shows the volume-time relationship of gas production at room temperature, indicating that the device can stably provide a gas source during operation. This dynamic gas generation process is further illustrated in Supplementary Movie S2. Optical images also show significant bubble generation in the solution reservoir after 100 s. Fig. 2I illustrates the displacement-time curve of the therapy module driven by the actuator's continuous operation. The actuator can consistently provide power, enabling the therapy module to move forward uniformly by 20 mm within 800 s. Fig. 2J demonstrates the actuator's propulsion capability, moving the therapy module forward by one turn (45 mm) within 100 s. The dynamic process is shown in Supplementary Movie S3.

All the materials used in the electrochemical pneumatic soft actuator are biocompatible, and the stable performance of the interdigitated electrodes, along with the actuator's excellent actuation capability, underscores its feasibility for in vivo operation, making future clinical translation possible.

### 2.3 Treatment module

**Fig. 3:**
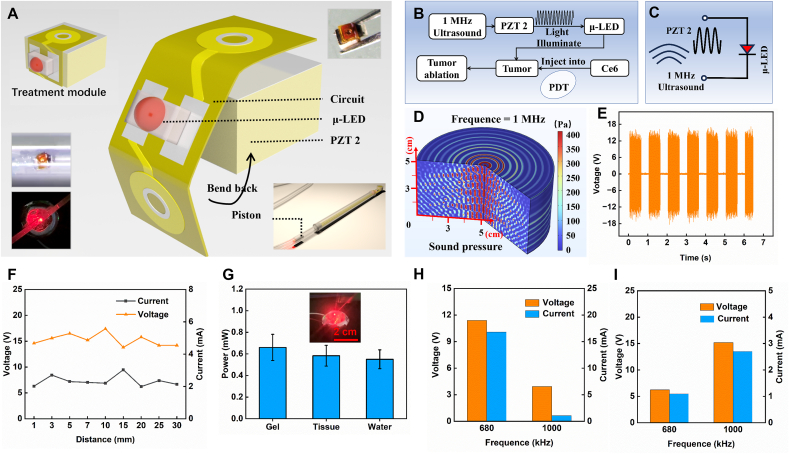
Structure and performance of the treatment module. (A) Exploded view of the treatment module. (B) Workflow of the treatment module in esophageal cancer therapy. (C) Energy harvesting circuit diagram of the treatment module. (D) Simulation of 1 MHz ultrasound propagation. (E) Open-circuit voltage output of PZT 2 at a 60 % duty cycle and 3 mm distance from the ultrasound source. (F) Short-circuit current and open-circuit voltage peak values of PZT 2 at distances of 1–30 mm from the ultrasound source. (G) Light power output of the μ-LED in the treatment module after receiving ultrasound in different media. (H) (I) Output performance of PZT 1 and PZT 2 at 680 kHz and 1 MHz, respectively, demonstrating that the actuator and treatment module operate without mutual interference.

Fig. 3 characterizes the structure and performance of the treatment module. Fig. 3A shows the structure and assembly process of the treatment module. A flexible circuit board is folded and soldered to the electrodes on both sides of PZT 2, with the other side connected to a μ-LED. The device is placed in the track of the actuator, resting solely on the outer end of the piston. The μ-LED (0.6 × 0.35 × 0.20 mm) and PZT 2 (1 × 1 × 0.6 mm, resonant frequency fc = 1 MHz) are small in size, ensuring the assembled module is compact and lightweight, allowing for easy movement within the actuator's track. Fig. 3B illustrates the working process of the treatment module. The μ-LED is pushed by the actuator near the tumor. PZT 2 receives 1 MHz ultrasound and converts the acoustic energy into electrical energy, lighting up the μ-LED. The 660 nm red light illuminates the tumor injected with photosensitizer, performing PDT on the tumor. Fig. 3C shows the circuit structure of the treatment module, where PZT 2 collects 1 MHz ultrasound, converts it into electrical energy, and transmits it to the μ-LED.

Fig. 3D presents a simulation of 1 MHz ultrasound propagation in water, indicating that ultrasound at this frequency can transmit with minimal attenuation through tissues, providing stable ultrasonic energy to the device. Fig. 3E shows the real-time open-circuit voltage output of PZT 2 at a distance of 7 mm from the ultrasound source, with a peak value reaching 15 V, sufficient to light up the μ-LED. Fig. 3F demonstrates that PZT 2 maintains stable output at various medium depths, with an open-circuit voltage peak around 15 V and a short-circuit current peak around 2.5 mA, ensuring stable operation at different depths. Fig. 3G evaluates the light power emitted by the device in different media: 0.65 mW in gel, 0.58 mW in tissue, and 0.55 mW in water, providing a stable light source for PDT. The dynamic process of ultrasound activating the treatment module to emit light is shown in Supplementary Movie S4. Fig. S3 (Supporting Information) shows the transmittance of different wavelengths of light through the silicone track of the actuator. The transmittance of 660 nm light is 86 %, indicating that most of the light produced by the therapeutic module can be used for PDT. Additionally, Fig. S4 (Supporting Information) describes the penetration capability of 660 nm red light. As the thickness of the pork tissue increases (1, 2, 3, and 4 mm), the transmittance of red light decreases (4.8 %, 2.38 %, 1.3 %, and 0.8 %, respectively). When the thickness exceeds 5 mm, the transmittance drops to below 0.05 %. These results highlight the limited penetration ability of light through tissue, making it challenging for traditional external illumination to penetrate the tissue effectively. However, our wireless system overcomes this limitation, effectively delivering the PDT light dose to the target area.

Fig. 3H and I depict the output performance of PZT 1 and PZT 2 at different ultrasound frequencies. PZT 1 is more sensitive to 680 kHz ultrasound, while PZT 2 is more sensitive to 1 MHz ultrasound. The different frequency selectivity of the two PZTs effectively prevent mis-activation in their respective working zones.

In terms of biological safety, we conducted further tests on the impact of the therapeutic module on local tissue (Fig. S12, Supporting Information). After operating continuously for 1 h, the muscle tissue containing the module showed a minimal increase of less than 2 °C (25.2 °C–27 °C), demonstrating that the system does not cause any thermal damage to the local tissue (Fig. S13, Supporting Information).

### 2.4 Efficacy of PDT on esophageal cancer cells in vitro

**Fig. 4:**
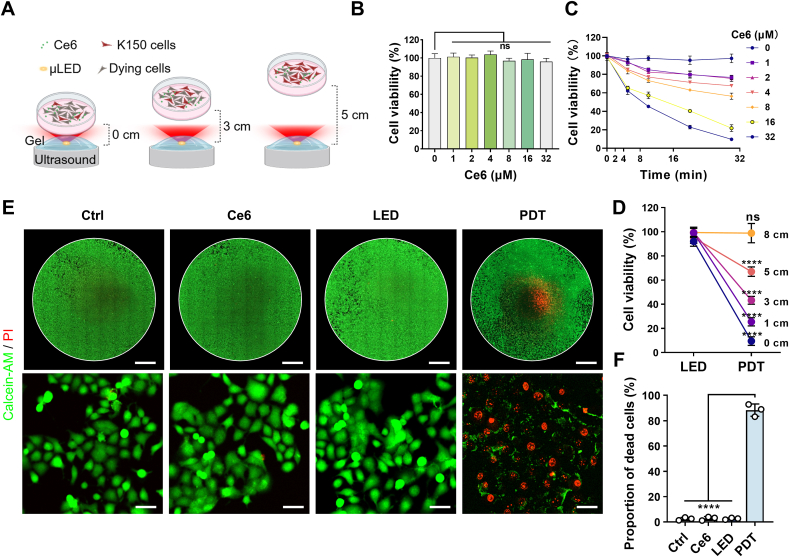
Significant cytotoxic impact of PDT on ESCC in vitro. (A) Schematic diagram of the PDT. (B) Cell viability of K150 under different concentrations of Ce6, *n* = 3. (C) The viability of K150 cells was assessed using the cell counting kit-8 (CCK-8) assay across various of Ce6 concentrations and exposure durations, *n* = 3. (D) The effect of PDT on the viability of K150 cells at varying treatment distances, *n* = 3. (E) WSI and microscopy imaging of Calcein-AM/PI staining in different treatment groups. Scar bars, 1000 μm for WSI and 50 μm for microscopy imaging. (F) Statistical analysis of dead cells proportion in different treatment groups, *n* = 3. Data are presented as mean ± SEM. Statistical analysis was conducted using ordinary one-way ANOVA with multiple comparisons, not significant (ns), *P* ≥ 0.05; ∗∗∗∗*P* ≤ 0.0001.

Fig. 4 illustrates the efficacy of PDT treatment module in eradicating esophageal cancer cells, as well as the impact of light source distance on PDT effectiveness in vitro. Fig. 4A illustrates the process of PDT conducted through the treatment module. The base layer comprises an ultrasound source with the treatment module positioned on ultrasound gel. When activated, the ultrasound triggers the μLED to illuminate, facilitating PDT. At the topmost layer, a cell plate containing the human esophageal squamous cell carcinoma (ESCC) cell line KYSE-150 (K150), pre-treated with the photosensitizer chlorin e6 (Ce6), is positioned for PDT. To assess whether the photosensitizer Ce6 exerts any effects on cells proliferation, we cultured the K150 with varying concentrations of Ce6 for 24 h. Our CCK-8 assay results confirmed that Ce6, even at a concentration of 32 μM, does not inhibit the viability of K150. (Fig. 4B). However, when Ce6 was present in conjunction with the μLED, PDT was effectively achieved. The efficacy of PDT was positively correlated with both the duration of illumination and the concentration of the photosensitizer (Fig. 4C). Specifically, at a Ce6 concentration of 16 μM and an illumination duration of 30 min, the cell viability of K150 was reduced to 23.66 %.

To evaluate the impact of light source distance (LSD) on PDT efficacy, we further measured cell viability following treatment at varying distances. Results indicated a marked decrease in viability (to 9.63 %) when cells were positioned close to the light source. In contrast, when the LSD was increasing to 8 cm, the viability of K150 cells remained largely unaffected (Fig. 4D). Additionally, the Calcein-AM/PI staining method provided further insight into PDT-induced cytotoxicity in the K150 cell line. Whole slide imaging (WSI) of cell plates revealed a concentrated red fluorescence signal at the center of the light source, indicative of a higher cell death rate in this region. As the distance from the center increased, the red fluorescence signal diminished, while green fluorescence, marking viable cells, became more prominent. Quantitative analysis of fluorescence images confirmed that PDT induced cell death in over 88.94 % of cells, underscoring the effectiveness of PDT at close proximity to the light source. (Fig. 4E and F).

During PDT, the cytotoxic agent singlet oxygen, produced via a type II photochemical reaction, is considered the primary mediator of PDT's biological effects [[66], [67], [68]]. To detect singlet oxygen generation, we employed 1,3-diphenylisobenzofuran (DPBF) as a specific probe. A decrease in DPBF's relative absorbance indicates an increased rate of photodegradation, reflecting elevated ROS production. We selected porphyrin as a standard photosensitizer and compared it with Ce6 (Fig. S10, Supporting Information). Under identical PDT conditions, DPBF absorbance decreased significantly with Ce6, while the absorbance change was negligible with porphyrin, indicating that Ce6 continuously generates singlet oxygen over prolonged irradiation, whereas porphyrin produces only trace amounts. These results demonstrate that Ce6 has a higher singlet oxygen generation efficiency compared to porphyrin. Furthermore, UV–visible absorption spectra confirmed that Ce6 exhibits stronger absorption in the red-light spectrum than porphyrin, making it more suitable for red-light-activated PDT.

To optimize light penetration depth, we selected a wavelength of 660 nm, as red light penetrates biological tissues more effectively than other wavelengths, such as ultraviolet and visible light. This allows it to reach several millimeters or deeper within tissue, enhancing the treatment effect. Furthermore, our implantable device effectively addresses the limitation of light penetration depth; by directly implanting the light source near the tumor, red light can reach areas inaccessible to external sources, thereby enhancing ROS generation.

**Fig. 5:**
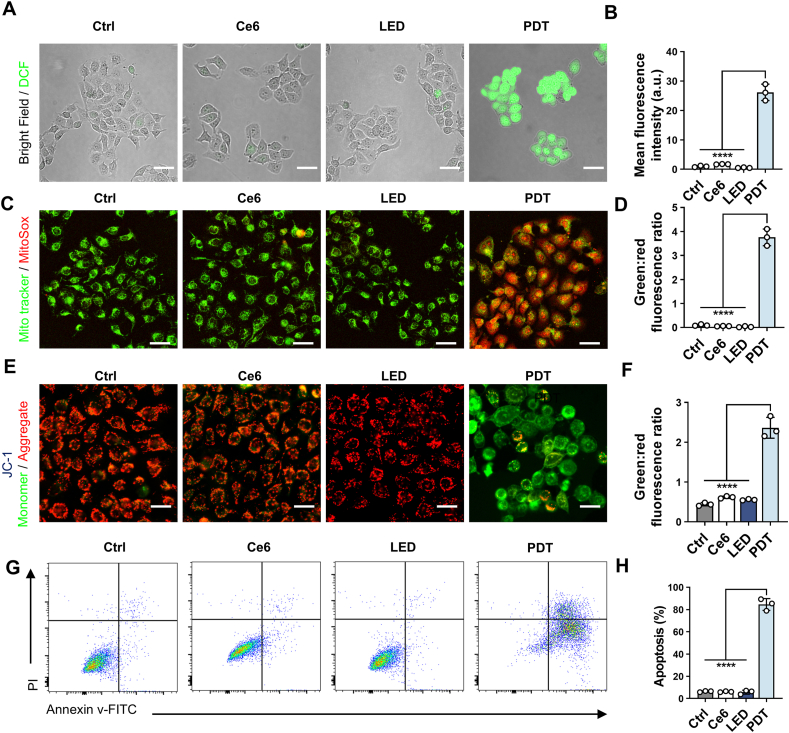
PDT significantly enhances the ROS generation and apoptosis in ESCC cells. (A) Representative images of ROS levels detected by DCFH-DA probe. Scar bars, 50 μm. (B) Statistical analysis of ROS in different treatment groups, *n* = 3. (C) Detection of mitochondrial superoxide expression by MitoSox red staining (red fluorescence indicates mitochondrial superoxide, green fluorescence marks the mitochondrion). Scar bars, 50 μm. (D) Quantitative analysis of mitochondrial superoxide, *n* = 3. (E) Fluorescence images of JC-1 staining (red fluorescence indicates JC-1 aggregates, green fluorescence indicates JC-1 monomers). Scar bars, 50 μm. (F) The corresponding statistical analysis of JC-1, *n* = 3. (G) Apoptosis detection by flow cytometry using the Annexin V-FITC/PI kit in different groups. (H) The corresponding statistical analysis of apoptosis, *n* = 3. Data are presented as mean ± SEM. Statistical analysis was conducted using ordinary one-way ANOVA with multiple comparisons, not significant (ns), *P* ≥ 0.05; *∗∗∗∗P* ≤ 0.0001. (For interpretation of the references to colour in this figure legend, the reader is referred to the Web version of this article.)

The efficiency of singlet oxygen generation is a critical factor in determining the effectiveness of PDT. To investigate the PDT yield, we employed the DPBF probe, and Fig. S11 reveals a notable reduction in the absorption intensity of DPBF's UV absorption spectrum after just 10 min of light exposure. This decrease provides strong evidence that our device generates a substantial yield of singlet oxygen during the PDT process. Furthermore, we utilized the DCFH-DA probe to quantitatively assess the ROS levels following PDT. The data indicated a marked increase in the mean fluorescence intensity (MFI) of ROS in the PDT group compared to the control group, highlighting the significant production of reactive oxygen species as a result of the treatment (Fig. 5A and B). To investigate the subcellular structures primarily responsible for ROS generation, we performed the MitoSOX staining to assess the expression levels of superoxide in the mitochondria. The experimental results showed that PDT group exhibited a significant increase in red fluorescence intensity, reflecting an elevated production of mitochondrial superoxide (Fig. 5C). As shown in Fig. 5D, the substantially higher green-to-red fluorescence ratio in the PDT group quantitatively supports this trend, indicating a significant increase in mitochondrial oxidative stress following PDT treatment. Further, we employed JC-1 fluorescence to detect mitochondrial membrane potential, shown in Fig. 5E and F. The green/red fluorescence ratio in the PDT group was 2.28, whereas other groups did not exceed 0.5 underscoring that PDT significantly promotes cancer cell apoptosis.

Additionally, flow cytometric analysis using an Annexin V/PI staining kit revealed that the PDT group exhibited the highest proportion of cells in both early and late stages of apoptosis (Fig. 5G and H). These results suggest that PDT induces significant oxidative stress and promotes apoptosis, effectively leading to the death of tumor cells.

### 2.5 In-vivo PDT treatment

**Fig. 6:**
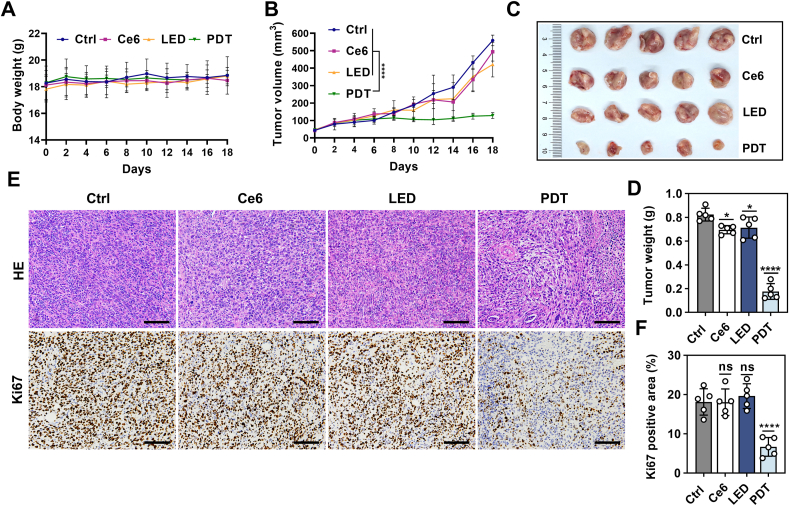
In vivo anti-tumor effect of the PDT treatment module. (A) Body weight of mice receiving various treatments, *n* = 5. (B) Average tumor growth curves for all groups, *n* = 5. (C) Tumor images collected from each group. (D) Average tumor mass collected from each group, *n* = 5. (E) Histological analysis of tumor tissue sections via H&E staining and Ki-67 immunohistochemical staining. Scale bar, 100 μm. (F) Quantitative analysis of Ki-67 immunohistochemical index, *n* = 5. All data are presented as mean ± SD, ∗*P* < 0.05, ∗∗∗*P* < 0.001, ∗∗∗∗*P* < 0.0001. Statistical analysis was conducted using ordinary one-way ANOVA with multiple comparisons.

Fig. 6 shows the in-vivo efficacy of the PDT treatment module. To establish a breast cancer a mouse model, 100,000 4T1 cells were subcutaneously implanted into Balb/c mouse. When the tumor volume reached about 50 mm³, the treatment module was implanted deep into the tumor tissue. Following intra-tumoral injection of 0.5 mg/kg Ce6 for 5 h, a wireless ultrasound probe was used to drive the μ-LED emission and generate photodynamic effects for 30 min per day over a period of 8 days. During this process, there was no significant difference in the body weight changes between the control or experimental groups (Fig. 6A), indicating the biocompatibility and safety of the treatment. Compared to the Ctrl group, the PDT group exhibited a significant trend of tumor suppression, demonstrating a strong inhibitory effect on tumor growth, while the tumors volume in the Ce6 and LED groups continued to grow rapidly (Fig. 6B).

After the 4T1 cells were implanted for 18 days, the subcutaneous tumors were excised to evaluate the efficacy of the treatment (Fig. 6C). The average tumor mass in the PDT group similarly decreased by 79.1 %, demonstrating the effectiveness of the therapy (Fig. 6D). In addition, we performed hematoxylin and eosin (H&E) staining and Ki67 immunohistochemical staining to assess the histological changes in the tumors (Fig. 6E). The H&E staining showed a significant reduction in tumor cell density and damage of the tumor stromal structure in the PDT group, while the PDT group showed the lowest positive area rate of Ki67 (Fig. 6F). These findings collectively suggest that the PDT achieved through the treatment module significantly suppresses tumor growth, underscoring its potential as a viable therapeutic strategy for cancer treatment. The morphology of major organs (Fig. S15, Supporting Information) indicates that our implantable treatment module has low side effects and high biocompatibility in vivo.

### 2.6 Biological safety Evaluation

**Fig. 7:**
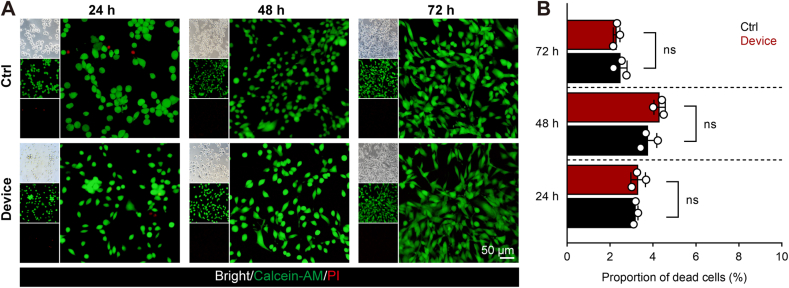
Evaluation of the biosafety of the integrated esophageal stent system. (A) Fluorescence microscopy images of Calcein-AM/PI staining after co-culturing the integrated stent system with NIH3T3 cells for 24, 48, and 72 h. (B) Statistical analysis of the proportion of cell death in different time groups, *n* = 3. Data are presented as mean ± SD. Unpaired *t*-test, not significant (ns), *P* ≥ 0.05.

Fig. 7 illustrates the biological safety of the integrated esophageal stent system. We co-cultured the device with mouse embryonic fibroblasts (NIH3T3) for 24, 48, and 72 h. Fluorescence microscopy imaging of Calcein-AM/PI staining showed that the stent could coexist with the cells for an extended period with minimal toxicity, as the proportion of cell apoptosis did not exceed 5 % (Fig. 7A and B).

In the experiments, the ultrasound intensity used was 407.42 mW/cm^2^, which is below the FDA-approved threshold (peak intensity of 720 mW/cm^2^) [69] to prevent damage to esophageal tissue from high-intensity ultrasound. Furthermore, histological analysis was conducted on the surrounding skin tissue and esophageal region to assess the safety of ultrasound exposure. The results showed that, compared to the control group, there were no significant morphological changes in the skin tissue and corresponding esophageal region following ultrasound irradiation (Fig. S14, Supporting Information), indicating that our ultrasound parameters are biologically safe.

## 3 Discussion

In this study, we propose a novel esophageal stent integrated with a wireless, battery-free, and movable PDT unit, designed for flexible, precise, and real-time treatment of esophageal tumors. This system enables ultrasound-based wireless control of the PDT light source position, providing a novel approach for treating esophageal cancer patients. However, several challenges remain in translating this technology into clinical practice. The primary obstacle is the issue of light penetration and diffusion in heterogeneous tumor environments. One of the main challenges in applying PDT in clinical settings is the limited tissue penetration of light, particularly in deeper tumor tissues. Furthermore, in heterogeneous tumor environments, due to varying tissue densities and compositions, light diffusion may be uneven, potentially leading to suboptimal activation of photosensitizers and incomplete tumor treatment.

To mitigate the limitations of light penetration, we utilized longer-wavelength red light, which is more tissue-penetrative and coincides with the absorption peak of Ce6. Additionally, we employed an implantable device to position the light source directly around the tumor. To ensure consistent treatment, we are considering the integration of photodetectors and fluorescence-based ROS sensors to monitor light distribution and ROS production.

Concerns about potential off-target ROS effects in healthy tissues are valid. To address this, we leverage the selective accumulation of Ce6 in tumor tissues. Upon systemic or local administration, Ce6 preferentially concentrates in cancer cells due to metabolic changes and increased blood flow in the tumor microenvironment [70,71]. Additionally, our system maximizes light delivery by positioning the μ-LED light source directly adjacent to the tumor, ensuring precise targeting of the treatment area. Furthermore, we are exploring the use of nanocarriers for the targeted delivery of photosensitizers, enabling controlled release and minimizing unintended damage to surrounding healthy tissues.

Regarding regulatory approval and safety, the clinical application of this novel therapeutic system will require extensive regulatory approval processes, including demonstrating the biocompatibility and safety of all system components. The μ-LEDs, soft actuators, piezoelectric transducers, and all electronic and structural components must undergo rigorous biocompatibility testing to ensure they do not induce inflammation, toxicity, or immune responses in vivo. Furthermore, the long-term stability and potential degradation of biocompatible packaging must be evaluated to prevent any adverse effects such as material wear or breakdown.

Finally, to enable widespread clinical adoption, the scalability of manufacturing this system must be addressed. The production of such a complex integrated system may face challenges related to cost, consistency, and reliability at large scales. It is crucial to establish standardized protocols for the assembly of the stent, μ-LEDs, actuators, and electronic components in a cost-effective and reproducible manner. Additionally, the integration of this system into existing clinical workflows must be considered to ensure ease of deployment and control.

In conclusion, this system offers a novel strategy for esophageal cancer treatment. Overcoming the identified challenges and further optimizing its components will be essential for enabling its clinical translation, potentially providing a new, targeted therapeutic option for managing complex tumor environments.

## 4 Conclusion

We have designed a wireless, battery-free, movable PDT unit for esophageal stents, enabling flexible and precise treatment of esophageal cancer. This system introduces a novel strategy utilizing an electrochemical pneumatic soft actuator to move PDT light sources in real-time to the vicinity of tumors, achieving precise and efficient treatment of esophageal tumors. The treatment module, characterized by its small size and wireless, battery-free operation, provides the light source for PDT therapy and moves frictionlessly within the track of the actuator. The flexible actuator adapts to various deformations, allowing it to spiral and cover a large area inside the esophageal stent, thereby extending the reach of PDT light sources. The actuator and treatment module exhibit different frequency selectivity to external ultrasound, enabling independent and interference-free operation. In vitro cell experiments have demonstrated the effective killing of tumor cells by PDT, with better efficacy observed at closer distances to the tumor, thereby establishing the practicality of this scaffold system. This innovative treatment approach holds promise for providing more effective and personalized treatment options for esophageal cancer patients, offering new ideas and methods for clinical intervention.

## 5 Materials and methods

### 5.1 Materials

PZT was purchased from SCH Technology Co., Ltd. The 660 nm μ-LEDs were obtained from Shenzhen Ruikoo Optoelectronics Technology Co., Ltd. (Cell experiment materials source).

### 5.2 Fabrication of treatment module

Customized PZT 2 (1 × 1 × 0.6 mm) was pre-cleaned with ethanol and deionized water. PZT 2 was soldered to a customized flexible micro-circuit board along with a 660 nm μ-LED. Optical image of PZT 2 is shown in Fig. S5 (Supporting Information), and scanning electron microscope (SEM) images are shown in Fig. S7 (Supporting Information). The circuit board layout is depicted in Fig. S8 (Supporting Information).

### 5.3 Fabrication of actuator

A flexible copper-clad PI sheet (Cu/PI/Cu, 12/12.5/12 μm) served as the substrate. Patterned transmission and finger electrodes were achieved on the substrate through exposure, development, and etching steps. Holes (300 μm in diameter) were drilled on the substrate, with inner walls copper-plated to ensure electrical connection between top and bottom electrodes. A 75 nm gold layer was chemically deposited on the finger electrodes to prevent oxidation in the presence of NaOH solution. Key electronic components and power supply units, including capacitors, μ-LEDs, diodes, and PZT 1, were assembled on the substrate through soldering. A 6 cm length, 2.5 cm inner diameter, and 3.5 mm outer diameter silicone tube served as the actuator's solution reservoir. A 22 cm length, 1.5 cm inner diameter, and 2.5 mm outer diameter silicone tube served as the delivery track. Both tubes were compactly connected using PDMS: curing agent (SYLGARD 184, Dow Corning, USA) mixed at a 10:1 mass ratio, applied to one end of the track, and connected to the solution reservoir, cured at 70 °C for 3 h. The electrodes were placed into the solution reservoir, and the other end was sealed with PDMS, simultaneously encapsulating the external circuit, cured at 70 °C for 3 h. A 50 mmol L^−1^ NaOH solution (Shanghai, Macklin Biochemical Co., Ltd., Shanghai, China) was injected into the solution reservoir from the end of the track using a 30 cm long syringe, followed by injecting vaseline at the bottom of the track to act as a piston to prevent leakage. The treatment module was inserted into the track and placed near the vaseline piston. Optical image of PZT 1 is shown in Fig. S6 (Supporting Information), SEM images in Fig. S7 (Supporting Information), and the circuit board layout in Fig. S9 (Supporting Information).

### 5.4 Characterization and measurement

The structure and morphology of materials were studied using a SEM (GeminiSEM 300, Germany). The piezoelectric output of the wireless power unit was measured using an oscilloscope (UTD4102C) and KEITHLEY (DMM7510). Finite element simulations of ultrasound were conducted using COMSOL. Measure the voltage-current curves using an electrochemical workstation (CHI600E). The volume of gas produced was determined by the height of the liquid column.

### 5.5 Cell culture

Human esophageal squamous cell carcinoma KYSE-150 cells were cultured in 1640 medium supplemented with 10 % fetal bovine serum and 1 % Penicillin/Streptomycin. Cells were then incubated at 37 °C in a humidified atmosphere containing 5 % CO_2_.

### 5.6 Ce6 toxicity assay

Using a 48-well plate, 10,000 cells per well were seeded and treated with 300 μl gradients of Ce6 (1, 2, 4, 6, 8, 16, and 32 μM) in culture medium. After 24 h of incubation, cell viability was assessed using the CCK-8 assay (ABclonal Technology, China).

### 5.7 Cell viability assay

In a 48-well plate, 10,000 cells per well were seeded. After incubating for 5 h in serum-free medium with a fixed Ce6 concentration of (0, 1, 2, 4, 6, 8, 16, and 32 μM) under 5 % CO_2_ at 37 °C, the medium was replaced with serum-containing complete medium. Cells treated with Ce6 were then exposed to μ-LED (660 nm, 25 mW) for varying durations (0, 5, 10, 20, 30, and 40 min), and the different distances form light source to the bottom of the cell plates (0, 1, 3, 5, and 8 cm). Cell viability was measured using the CCK-8 assay after 24 h of incubation.

### 5.8 Live/dead assay

K150 cells (100,000 per well) were seeded in 3.5 cm dishes and subjected to control, LED, Ce6, and PDT. After 24 h, the cells were stained with 500 μL of Calcein-AM/PI working solution (Beyotime Biotechnology, China) and incubated at 37 °C in the dark for 30 min. Following staining, WSI was performed using a cell imaging multi-mode microplate reader (Agilent BioTek Cytation 5, America). Additionally, K150 cells were also seeded in 48-well cell culture plates, treated and stained under the same conditions, and then imaged using a confocal fluorescence microscope (Nikon, Japan).

### 5.9 Measurement of intracellular ROS levels

Cells (100,000 per well) were seeded in 3.5 cm dishes. Cells from control, LED, Ce6, and PDT groups were incubated with DCFH-DA staining solution (Beyotime Biotechnology, China) at 37 °C for 20 min. ROS levels were measured using a confocal microscope (Nikon, Japan) with excitation and emission wavelengths set at 488 and 525 nm, respectively.

### 5.10 Mitochondrial superoxide detection

Cells (100,000 per well) were seeded in a 6-well plate and subjected to PDT for 1 h. The mitochondria were stained with 5 μM MitoSOX Red (Beyotime, China) at 37 °C for 20 min to label mitochondrial superoxide. Following staining, the cells were washed twice with PBS. Subsequently, the mitochondria were further labeled with 0.1 μM Mito-Tracker Green (Beyotime, China) at 37 °C for 20 min. After another round of washing, the samples were observed using a laser scanning confocal microscope.

### 5.11 Apoptosis assay

Cells (150,000 per well) were seeded in 6-well plates and cultured for 48 h in control, LED, Ce6, and PDT conditions. Apoptosis levels were measured using the Annexin V-FITC/PI Apoptosis Kit (4abio tech, China).

### 5.12 Mitochondrial membrane potential assay

Cells (100,000 per well) were seeded in 3.5 cm dishes. Divide the cells into four groups: control, LED, Ce6, and PDT. After 24 h of cultivation, the mitochondrial membrane potential was measured by the JC-1staining kit (Beyotime Biotechnology, China).

### 5.13 In vivo anticancer effect of PDT

Animal experiments were conducted in accordance with the Principles of Laboratory Animal Care (People's Republic of China) and all animal experiments were conducted with ethic approval (SCCHEC-04-2023-026).

In this experiment, the 4T1 mouse breast cancer cell line was used for in vivo tumor-bearing treatment. First, 100,000 4T1 cells were subcutaneously injected into mice to establish the tumor model. Treatment was initiated when the tumor volume reached approximately 50 mm³. During the treatment period, the body weight and tumor volume of the mice were measured and recorded every two days. The treatment was administered daily for 8 consecutive days. After treatment, the mice were monitored, and tumor volume changes were recorded for an additional 10 days to complete the observation period.

### 5.14 Biocompatibility evaluation of treatment formulations

Biocompatibility Evaluation of Treatment Formulations NIH3T3 cells were cultured in DMEM supplemented with 10 % FBS and 1 % P/S for 24 h. Using a 96-well plate, 100,000 cells per well were seeded. Integrated scaffold slices were separately co-cultured with normal NIH3T3 cells for 24, 48, and 72 h, followed by Calcein-AM/PI staining to assess cell viability and death.

### 5.15 Safety evaluation of ultrasound intensity

Eight-week-old female SD rats underwent neck hair removal, followed by anesthesia. Gel was applied to the corresponding sites of the cervical and thoracic esophagus, and ultrasound treatment (with specified power) was administered daily for 20 min per session over a period of seven days. Afterward, the esophagus and corresponding skin were harvested for histological analysis using H&E staining to evaluate structural changes.

### 5.16 Statistical analysis

The ordinary one-way ANOVA with multiple comparisons or unpaired *t*-test was used to perform statistical analysis using GraphPad Prism 9 (GraphPad Software, Inc., California, USA). Statistically significant was concluded at ∗ P < 0.05, ∗∗ P < 0.01, ∗∗∗ P < 0.001, ∗∗∗∗ P < 0.0001. Data are presented as mean ± SD or mean ± SEM.

## CRediT authorship contribution statement

**Qian Han:** Writing – original draft, Investigation. **Pingjin Zou:** Writing – original draft, Investigation. **Xianhao Wei:** Writing – original draft, Investigation. **Junyang Chen:** Investigation. **Xiaojiao Li:** Investigation. **Li Quan:** Investigation. **Ranlin Wang:** Investigation. **Lili Xing:** Supervision, Funding acquisition, Conceptualization. **Xinyu Xue:** Writing – review & editing, Supervision, Funding acquisition, Conceptualization. **Yi Zhou:** Writing – review & editing, Funding acquisition, Conceptualization. **Meihua Chen:** Writing – review & editing, Supervision, Funding acquisition, Conceptualization.

## Declaration of competing interest

The authors declare that they have no known competing financial interests or personal relationships that could have appeared to influence the work reported in this paper.

## Data Availability

Data will be made available on request.

## References

[1] Sung H., Ferlay J., Siegel R.L., Laversanne M., Soerjomataram I., Jemal A., Bray F. (2021). Global cancer statistics 2020: GLOBOCAN estimates of incidence and mortality worldwide for 36 cancers in 185 countries. CA A Cancer J. Clin..

[2] Bray F., Ferlay J., Soerjomataram I., Siegel R.L., Torre L.A., Jemal A. (2018). Global cancer statistics 2018: GLOBOCAN estimates of incidence and mortality worldwide for 36 cancers in 185 countries. CA A Cancer J. Clin..

[3] Ferlay J., Colombet M., Soerjomataram I. (2021). Cancer statistics for the year 2020: an overview. Int. J. Cancer.

[4] Liu C.-Q., Ma Y.-L., Qin Q., Wang P.-H., Luo Y., Xu P.-F. (2023). Epidemiology of esophageal cancer in 2020 and projections to 2030 and 2040. Thorac Cancer.

[5] D'Journo X., Thomas P. (2014). Current management of esophageal cancer. J. Thorac. Dis..

[6] Watanabe M., Otake R., Kozuki R. (2020). Recent progress in multidisciplinary treatment for patients with esophageal cancer. Surg. Today.

[7] Cummings D., Wong J., Palm R., Hoffe S., Almhanna K., Vignesh S. (2021). Epidemiology, diagnosis, staging and multimodal therapy of esophageal and gastric tumors. Cancers.

[8] Moaven O., Wang T.N. (2019). Combined modality therapy for management of esophageal cancer: current approach based on experiences from east and west. Surg. Clin..

[9] He S., Xu J., Liu X., Zhen Y. (2021). Advances and challenges in the treatment of esophageal cancer. Acta Pharm. Sin. B.

[10] Yura M., Koyanagi K., Hara A., Hayashi K., Tajima Y., Kaneko Y., Fujisaki H., Hirata A., Takano K., Hongo K. (2021). Unresectable esophageal cancer treated with multiple chemotherapies in combination with chemoradiotherapy: a case report. World J Clin Cases.

[11] Ikeda G., Yamamoto S., Kato K. (2022). The safety of current treatment options for advanced esophageal cancer after first-line chemotherapy. Expert Opin Drug Saf.

[12] Dolmans D., Fukumura D., Jain R.K. (2003). Photodynamic therapy for cancer. Nat. Rev. Cancer.

[13] Li X.S., Lovell J.F., Yoon J., Chen X.Y. (2020). Clinical development and potential of photothermal and photodynamic therapies for cancer. Nat. Rev. Clin. Oncol..

[14] Hopper C. (2000). Photodynamic therapy: a clinical reality in the treatment of cancer. Lancet Oncol..

[15] Dougherty T.J., Gomer C.J., Henderson B.W., Jori G., Kessel D., Korbelik M., Moan J., Peng Q. (1998). Photodynamic therapy. J Natl Cancer Inst.

[16] MacDonald I.J., Dougherty T.J. (2001). Basic principles of photodynamic therapy. J. Porphyr. Phthalocyanines.

[17] Oleinick N.L., Morris R.L., Belichenko T. (2002). The role of apoptosis in response to photodynamic therapy: what, where, why, and how. Photochem. Photobiol. Sci..

[18] Henderson B.W., Dougherty T.J. (1992). How does photodynamic therapy work. Photochem. Photobiol..

[19] Berr F. (2004). Photodynamic therapy for cholangiocarcinoma. Semin. Liver Dis..

[20] Marks C.E., Thomas S.M., Greenup R.A., Fayanju O.M., McDuff S., Kimmick G., Hwang E.S., Plichta J.K. (2020). Surgical management of the axilla in elderly women with node-positive breast cancer. J. Surg. Res..

[21] Arenas M., Fernandez-Arroyo S., Rodriguez-Tomas E., Sabater S., Murria Y., Gascon M., Amillano K., Mele M., Camps J., Joven J. (2020). Effects of radiotherapy on plasma energy metabolites in patients with breast cancer who received neoadjuvant chemotherapy. Clin. Transl. Oncol..

[22] Ethirajan M., Chen Y.H., Joshi P., Pandey R.K. (2011). The role of porphyrin chemistry in tumor imaging and photodynamic therapy. Chem. Soc. Rev..

[23] Lou P.J., Jager H.R., Jones L., Theodossy T., Bown S.G., Hopper C. (2004). Interstitial photodynamic therapy as salvage treatment for recurrent head and neck cancer. Br. J. Cancer.

[24] Kim M.M., Darafsheh A. (2020). Light sources and dosimetry techniques for photodynamic therapy. Photochem. Photobiol..

[25] Kulik M., Nedelcu C., Martin F., Lebdai S., Rousselet M.C., Azzouzi A.R., Aube C. (2014). Post-treatment MRI aspects of photodynamic therapy for prostate cancer. Insights Imaging.

[26] Kim A., Zhou J.W., Samaddar S., Song S.H., Elzey B.D., Thompson D.H., Ziaie B. (2019). An implantable ultrasonically-powered micro-light-source (μLight) for photodynamic therapy. Sci. Rep..

[27] Idris N.M., Gnanasammandhan M.K., Zhang J., Ho P.C., Mahendran R., Zhang Y. (2012). In vivo photodynamic therapy using upconversion nanoparticles as remote-controlled nanotransducers. Nat Med.

[28] Choi M., Choi J.W., Kim S., Nizamoglu S., Hahn S.K., Yun S.H. (2013). Light-guiding hydrogels for cell-based sensing and optogenetic synthesis in vivo. Nat. Photonics.

[29] Bansal A., Yang F.Y., Xi T., Zhang Y., Ho J.S. (2017). In vivo wireless photonic photodynamic therapy. Proc. Natl. Acad. Sci. U. S. A..

[30] Arami H., Kananian S., Khalifehzadeh L., Patel C.B., Chang E., Tanabe Y., Zeng Y.T., Madsen S.J., Mandella M.J., Natarajan A., Peterson E.E., Sinclair R., Poon A.S.Y., Gambhir S.S. (2022). Remotely controlled near-infrared-triggered photothermal treatment of brain tumours in freely behaving mice using gold nanostars. Nat. Nanotechnol..

[31] Guo H.W., Lin L.T., Chen P.H., Ho M.H., Huang W.T., Lee Y.J., Chiou S.H., Hsieh Y.S., Dong C.Y., Wang H.W. (2015). Low-fluence rate, long duration photodynamic therapy in glioma mouse model using organic light emitting diode (OLED), Photo Photodyn. Ther.

[32] Yamagishi K., Kirino I., Takahashi I., Amano H., Takeoka S., Morimoto Y., Fujie T. (2019). Tissue-adhesive wirelessly powered optoelectronic device for metronomic photodynamic cancer therapy. Nat. Biomed. Eng..

[33] Kirino I., Fujita K., Sakanoue K., Sugita R., Yamagishi K., Takeoka S., Fujie T., Uemoto S., Morimoto Y. (2021). Metronomic photodynamic therapy using an implantable LED device and orally administered 5-aminolevulinic acid. Sci. Rep..

[34] Liu Z., Xu L.L., Zheng Q., Kang Y., Shi B.J., Jiang D.J., Li H., Qu X.C., Fan Y.B., Wang Z.L., Li Z. (2020). Human motion driven self-powered photodynamic system for long-term autonomous cancer therapy. ACS Nano.

[35] Takahashi H., Arimura Y., Okahara S. (2015). A randomized controlled trial of endoscopic steroid injection for prophylaxis of esophageal stenoses after extensive endoscopic submucosal dissection. BMC Gastroenterol..

[36] Isomoto H., Yamaguchi N., Nakayama T. (2011). Management of esophageal stricture after complete circular endoscopic submucosal dissection for superficial esophageal squamous cell carcinoma. BMC Gastroenterol..

[37] László Szapáry B.T., Nelli Farkas K.M., Lajos Szakó Á.M. (2018). Intralesional steroid is beneficial in benign refractory esophageal strictures: a meta-analysis. World J. Gastroenterol..

[38] Lee J.G., Lieberman D. (1997). Endoscopic palliation for esophageal cancer. Dig. Dis..

[39] Sharma P., Kozarek R. (2010). Practice Parameters Committee of American College of G., Role of esophageal stents in benign and malignant diseases. Am. J. Gastroenterol..

[40] Hindy P., Hong J., Lam-Tsai Y. (2012). A comprehensive review of esophageal stents. Gastroenterol. Hepatol..

[41] Spaander M.C., Baron T.H., Siersema P.D. (2016). Esophageal stenting for benign and malignant disease: European Society of Gastrointestinal Endoscopy (ESGE) clinical guideline. Endoscopy.

[42] Messmann H., Holstege A., Szeimies R.M., Lock G., Bown S.G., Schölmerich J. (1995). Photodynamic therapy: a safe and effective treatment for tumor overgrowth in patients with oesophageal cancer and metal stents. Endoscopy.

[43] Katsu A., Yanagi M., Yoshioka M., Motoda N., Takata H., Kono H., Kimata R., Hamasaki T., Kondo Y. (2024). Preoperative rapid growth of inferior vena cava tumor thrombus in renal cell carcinoma. IJU Case Rep.

[44] Abrahamsson L., Czene K., Hall P. (2015). Breast cancer tumour growth modelling for studying the association of body size with tumour growth rate and symptomatic detection using case-control data. Breast Cancer Res..

[45] Sulciner M.L., Serhan C.N., Gilligan M.M., Mudge D.K., Chang J., Gartung A., Lehner K.A., Bielenberg D.R., Schmidt B., Dalli J. (2018). Resolvins suppress tumor growth and enhance cancer therapy. J. Exp. Med..

[46] Boulas K.A., Paraskeva A., Triantafyllidis A., Hatzigeorgiadis A. (2020). A case of massive primary tumor growth in the immediate postoperative period. Clin Case Rep.

[47] Zhang P., He H., Bai Y., Liu W., Huang L. (2020). Dexmedetomidine suppresses the progression of esophageal cancer via miR-143-3p/epidermal growth factor receptor pathway substrate 8 axis. Anti Cancer Drugs.

[48] Fong L.Y.Y., Zhang L., Jiang Y., Farber J.L. (2005). Dietary zinc modulation of COX-2 expression and lingual and esophageal carcinogenesis in rats. JNCI: Journal of the National Cancer Institute.

[49] Zhou S., Zhang F., Chen X., Jun J., Jing X., Wei D., Xia Y., Zhou Y., Xiao X., Jia R., Jia R. (2016). miR-100 suppresses the proliferation and tumor growth of esophageal squamous cancer cells via targeting CXCR7. Oncol. Rep..

[50] Zhang F., Yang Z., Cao M., Xu Y., Li J., Chen X., Gao Z., Xin J., Zhou S., Zhou Z., Yang Y., Sheng W., Zeng Y. (2014). MiR-203 suppresses tumor growth and invasion and down-regulates MiR-21 expression through repressing Ran in esophageal cancer. Cancer Lett..

[51] Ashuri T., Armani A., Jalilzadeh Hamidi R., Reasnor T., Ahmadi S., Iqbal K. (2020). Biomedical soft robots: current status and perspective. Biomed Eng Lett.

[52] Shepherd R.F. (2011). Multigait soft robot. Proc Natl Acad Sci..

[53] Cianchetti Matteo, Ranzani Tommaso, Gerboni Giada, Nanayakkara Thrishantha (2014). Soft robotics technologies to address shortcomings in today's minimally invasive surgery: the STIFF-FLOP approach. Soft Robot..

[54] Rossiter J., Hauser H. (2016). Soft robotics - the next industrial revolution? [Industrial activities]. IEEE Robot. Autom. Mag..

[55] Fan D., Liu H., Wang T., Zhu R., Wang H. (2023).

[56] Jung Y., Kwon K., Lee J. (2024). Untethered soft actuators for soft standalone robotics. Nat. Commun..

[57] Walker J., Zidek T., Harbel C., Yoon S., Strickland F.S., Kumar S., Shin M. (2020). Soft robotics: a review of recent developments of pneumatic soft actuators. Actuators.

[58] Jung Y., Kwon K., Lee J. (2024). Untethered soft actuators for soft standalone robotics. Nat. Commun..

[59] Zhong T., Yi H., Gou J. (2024). A wireless battery-free eye modulation patch for high myopia therapy. Nat. Commun..

[60] Ai T., Xing L., Jian L., Xu S., Minhua T., Li Z., Yue C. (2014). The biological safety evaluation of a newly developed silicone rubber for inflatable silastic prosthesis. Hua xi kou qiang yi xue za zhi.

[61] Sun Y., Fu H., Xu Y., Chen T., Liu Z., Liu X., Bing W. (2024). Study on hemostatic and antibacterial properties of modified silicone rubber sponge. React. Funct. Polym..

[62] Bélanger M.C., Marois Y. (2001). Hemocompatibility, biocompatibility, inflammatory and in vivo studies of primary reference materials low-density polyethylene and polydimethylsiloxane: a review. J. Biomed. Mater. Res..

[63] Baek J.Y., Kwon G.H., Kim J.Y., Cho J.H., Lee S.H., Sun K., Lee S.H. (2007). Stable deposition and patterning of metal layers on the PDMS substrate and characterization for the development of the flexible and implantable micro electrode. Solid State Phenom..

[64] Losi P., Briganti P., Magera E., Spiller A., Ristori D., Battolla C., Balderi B., Kull M., Balbarini S., Stefano A.D., Soldani R., Giorgio G. (2010). Tissue response to poly(Ether)Urethane-polydimethylsiloxane-fibrin composite scaffolds for controlled delivery of pro-angiogenic growth factors. Biomaterials.

[65] Zhong T., Yi H., Gou J. (2024). A wireless battery-free eye modulation patch for high myopia therapy. Nat. Commun..

[66] Przygoda M., Bartusik-Aebisher D., Dynarowicz K., Cieślar G., Kawczyk-Krupka A., Aebisher D. (2023). Cellular mechanisms of singlet oxygen in photodynamic therapy. Int. J. Mol. Sci..

[67] Mehraban N., Freeman H.S. (2015). Developments in PDT sensitizers for increased selectivity and singlet oxygen production. Mater.

[68] Zhu W., Gao Y.-H., Liao P.-Y., Chen D.-Y., Sun N.-N., Nguyen Thi P.A., Yan Y.-J., Wu X.-F., Chen Z.-L. (2018). Comparison between porphin, chlorin, and bacteriochlorin derivatives for photodynamic therapy: synthesis, photophysical properties, and biological activity. Eur. J. Med. Chem..

[69] Harris G., Services H. (2008). Guidance for industry and FDA staff information for manufacturers seeking marketing clearance of diagnostic ultrasound systems and transducers. Food Drug Admin..

[70] Hak A., Ali M.S., Sankaranarayanan S.A., Shinde V.R., Rengan A.K. (2023). Chlorin e6: A Promising Photosensitizer in Photo-Based Cancer Nanomedicine. ACS Appl. Bio Mater..

[71] Kou J., Dou D., Yang L. (2017). Porphyrin photosensitizers in photodynamic therapy and its applications. Oncotarget.

