# Supplementary material for: An esophageal stent integrated with wireless battery-free movable photodynamic-therapy unit for targeted tumor treatment
Source: Mater Today Bio. 2024 Dec 9;30:101394. doi: 10.1016/j.mtbio.2024.101394 (PMC11697610; doi:10.1016/j.mtbio.2024.101394)
Supplement: Multimedia component 1 [file mmc1.docx]

Supporting Information

**An Esophageal Stent Integrated with Wireless Battery-Free Movable Photodynamic-Therapy Unit for Targeted Tumor Treatment**

Qian Han ^a,c, #^, Pingjin Zou ^b, #^, Xianhao Wei ^a, #^, Junyang Chen ^b^, Xiaojiao Li ^a^, Li Quan ^d^, Ranlin Wang ^e^, Lili Xing ^a^, Xinyu Xue ^a,^ *, Yi Zhou ^f,^ *, Meihua Chen ^b,^ *

^a^ School of Physics, University of Electronic Science and Technology of China, Chengdu, 611731, China.

^b^ Department of Radiation Oncology, Radiation Oncology Key Laboratory of Sichuan Province, Sichuan Clinical Research Center for Cancer, Sichuan Cancer Hospital & Institute, Sichuan Cancer Center, Affiliated Cancer Hospital of University of Electronic Science and Technology of China, Chengdu, 610041, China.

^c^ School of Medicine, University of Electronic Science and Technology of China, Chengdu, 610054, China.

^d^ Chengdu University of Traditional Chinese Medicine, Chengdu, 610032, China.

^e^ Department of Endoscopy, Sichuan Clinical Research Center for Cancer, Sichuan Cancer Hospital & Institute, Sichuan Cancer Center, Affiliated Cancer Hospital of University of Electronic Science and Technology of China, Chengdu, 610041, China.

^f^ Department of Abdominal Oncology, Sichuan Clinical Research Center for Cancer, Sichuan Cancer Hospital & Institute, Sichuan Cancer Center, Affiliated Cancer Hospital of University of Electronic Science and Technology of China, Chengdu, 610041, China.

# These authors contributed equally to this work.

* Corresponding author Email: xuexinyu@uestc.edu.cn (X.X.); doctorzhouyi@163.com (Y.Z.); chenmeihua@scszlyy.org.cn (M.C.).


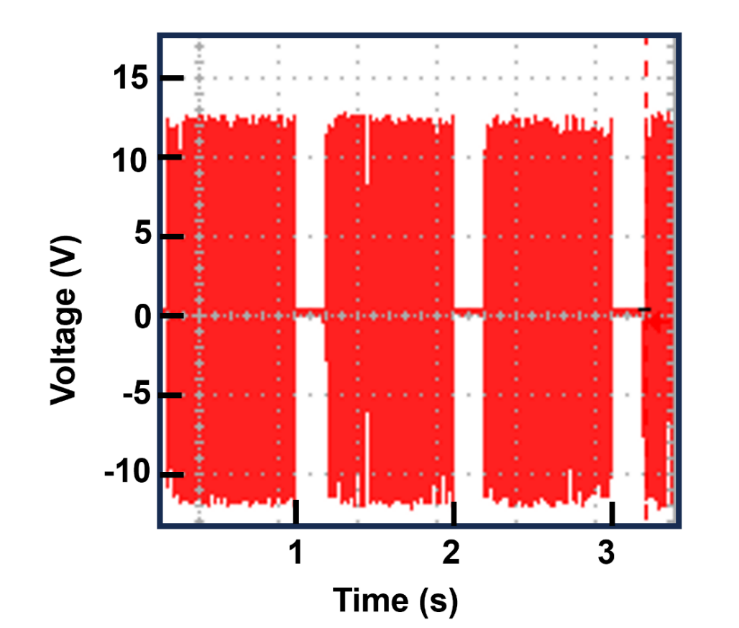


**Figure S1**. Voltage output of PZT 1 at an ultrasound duty cycle of 70%.





**Figure S2**. Current output of PZT 1 at an ultrasound duty cycle of 100%.


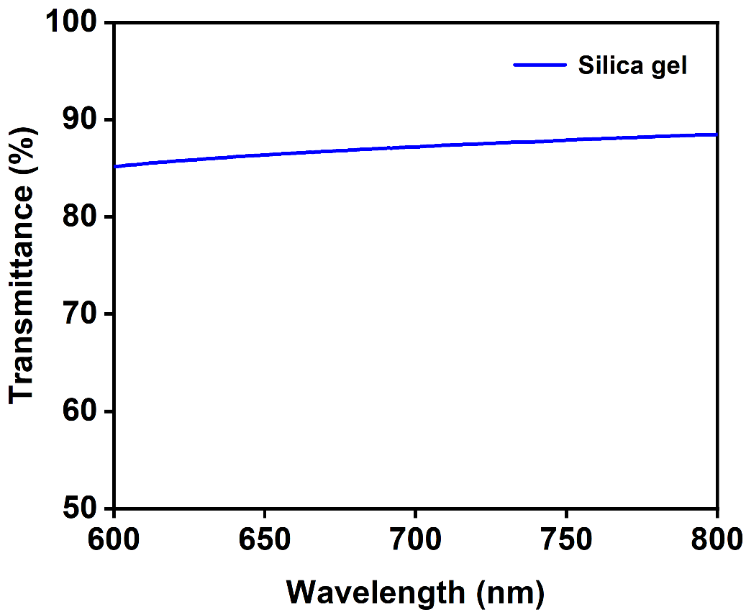


**Figure S3**. The transmittance of PDMS for different wavelengths of light


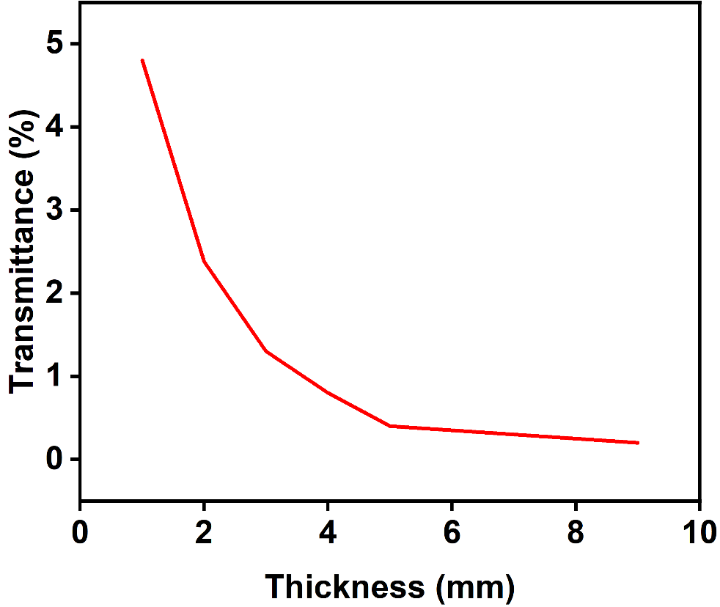


**Figure S4**. Tissue transmittance at varying thickness.


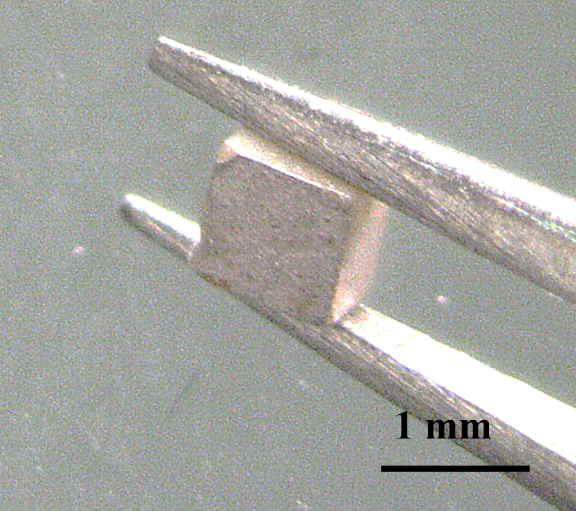


**Figure S5**. Optical image of PZT 2.


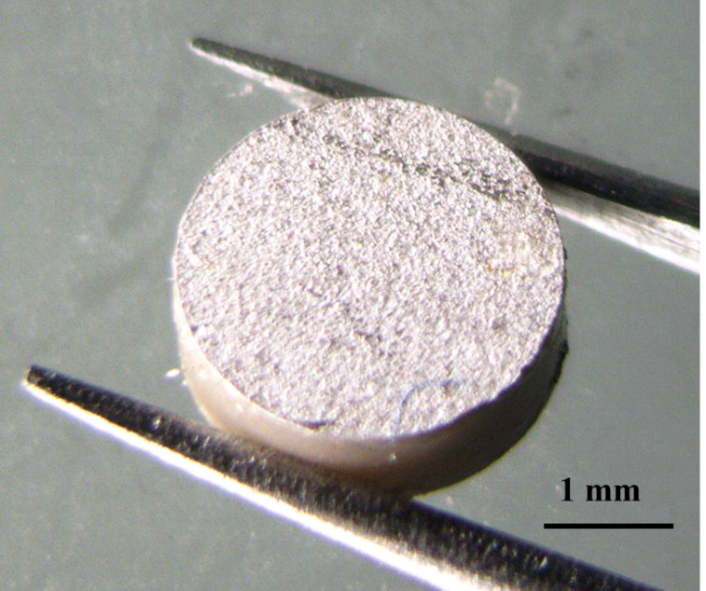


**Figure S6**. Optical image of PZT 1.


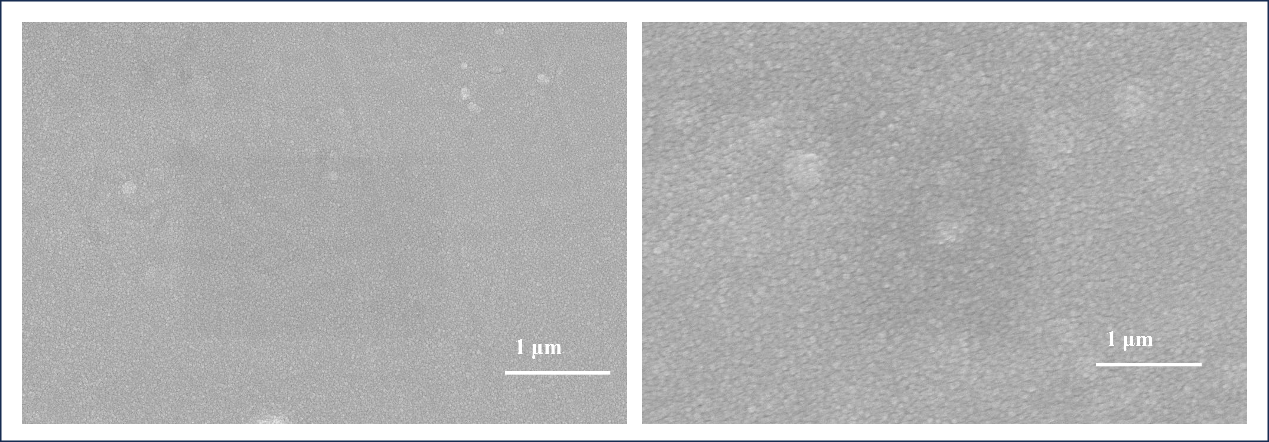


**Figure S7**. SEM of PZT.


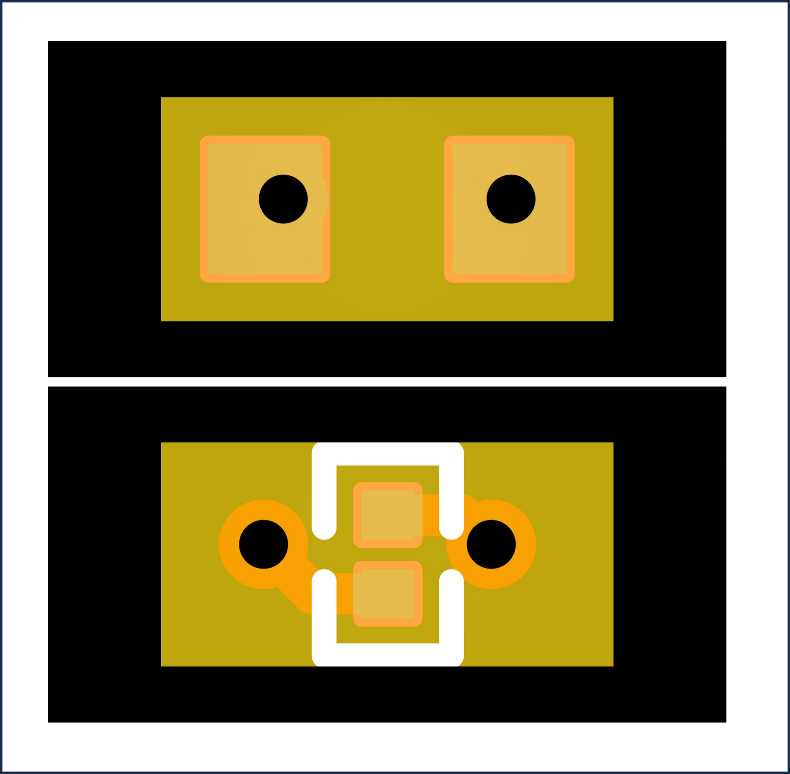


**Figure S8**. Circuit board diagram of the treatment module


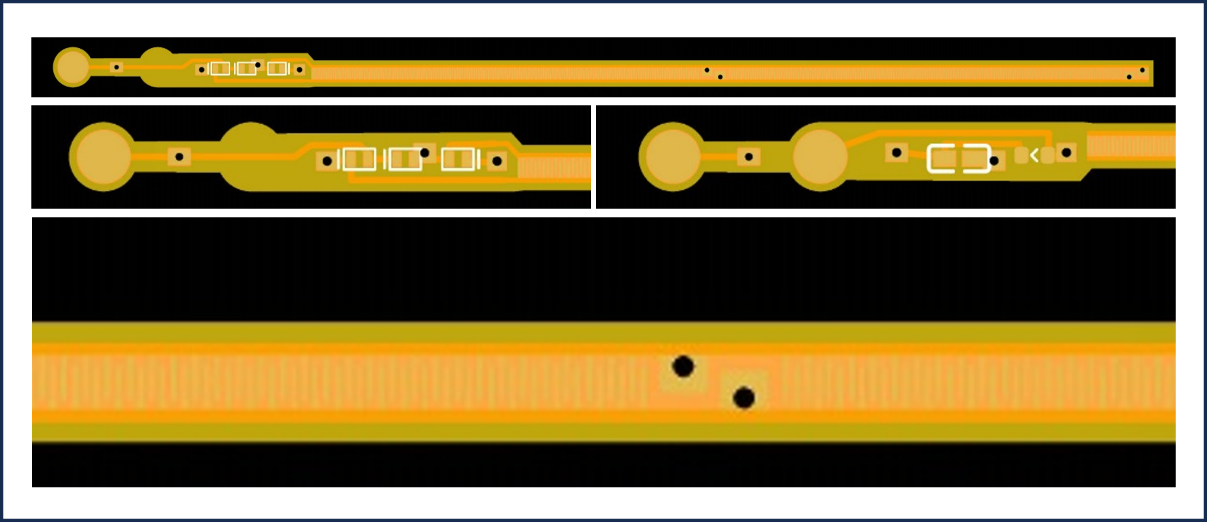


**Figure S9**. Circuit board diagram of the electrochemical brake


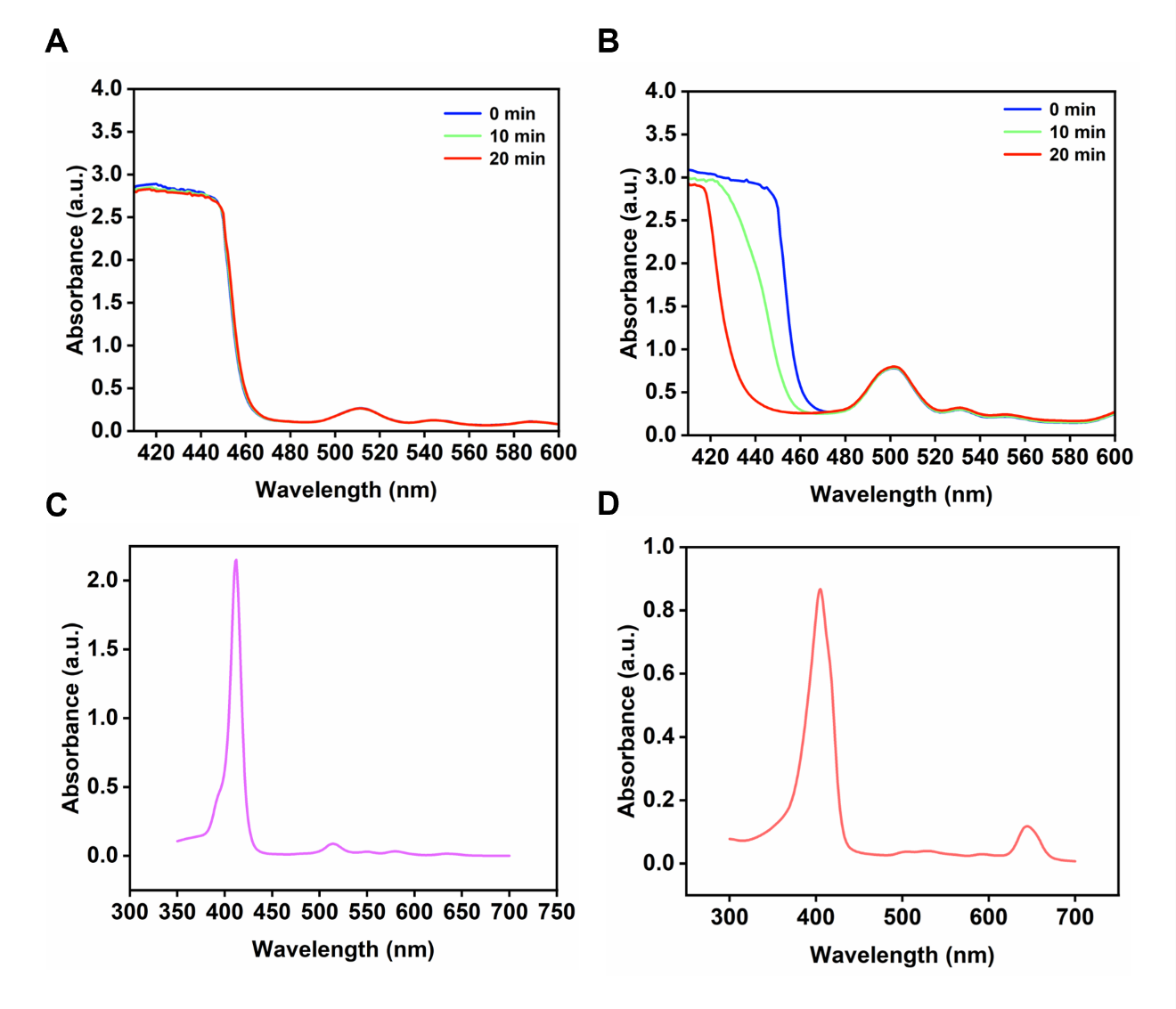


**Figure S10.** After the addition of (a) porphyrin, changes in the UV absorption spectrum of the DPBF probe; (b) Ce6, changes in the UV absorption spectrum of the DPBF probe; (c) UV absorption spectrum of porphyrin; (d) UV absorption spectrum of Ce6.





**Figure S11.** Changes in the UV absorption spectrum of DPBF+Ce6 after PDT.


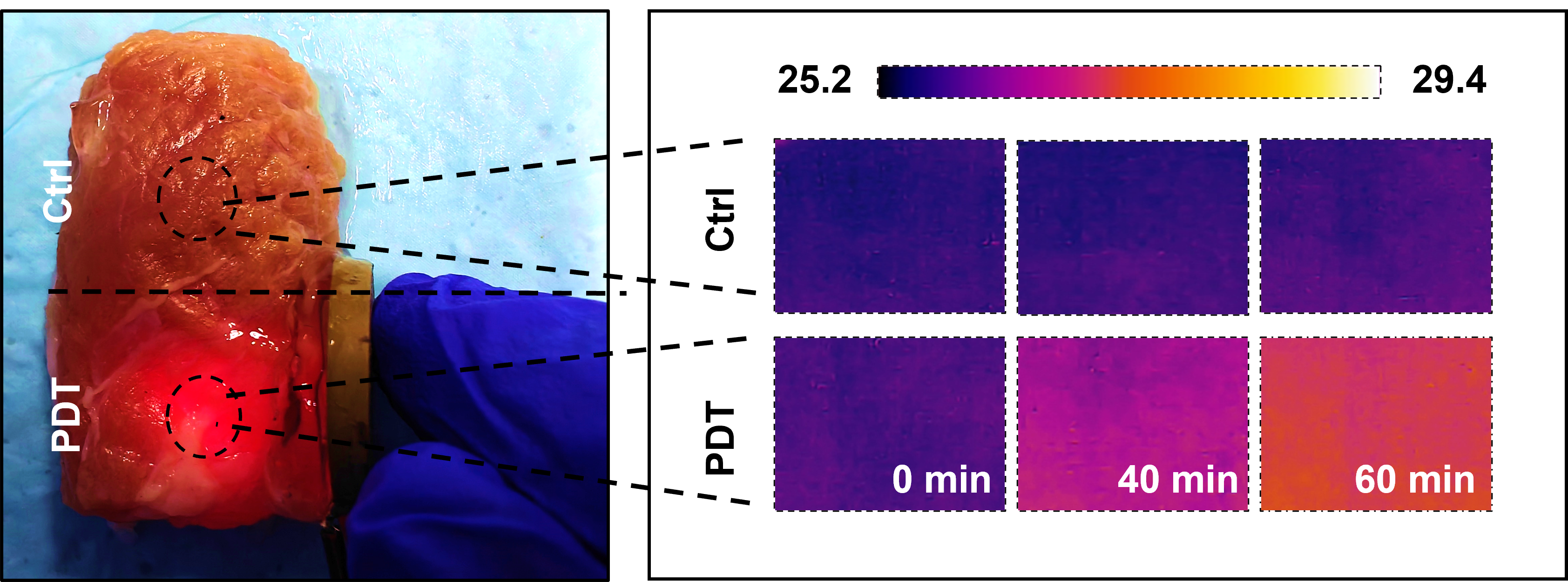


**Figure S12.** Temperature measurement diagram of the ultrasound probe and thermal imaging of local skin tissue.





**Figure S13.** The temperature change curve of local muscle tissue after one hour of therapeutic tablet operation.


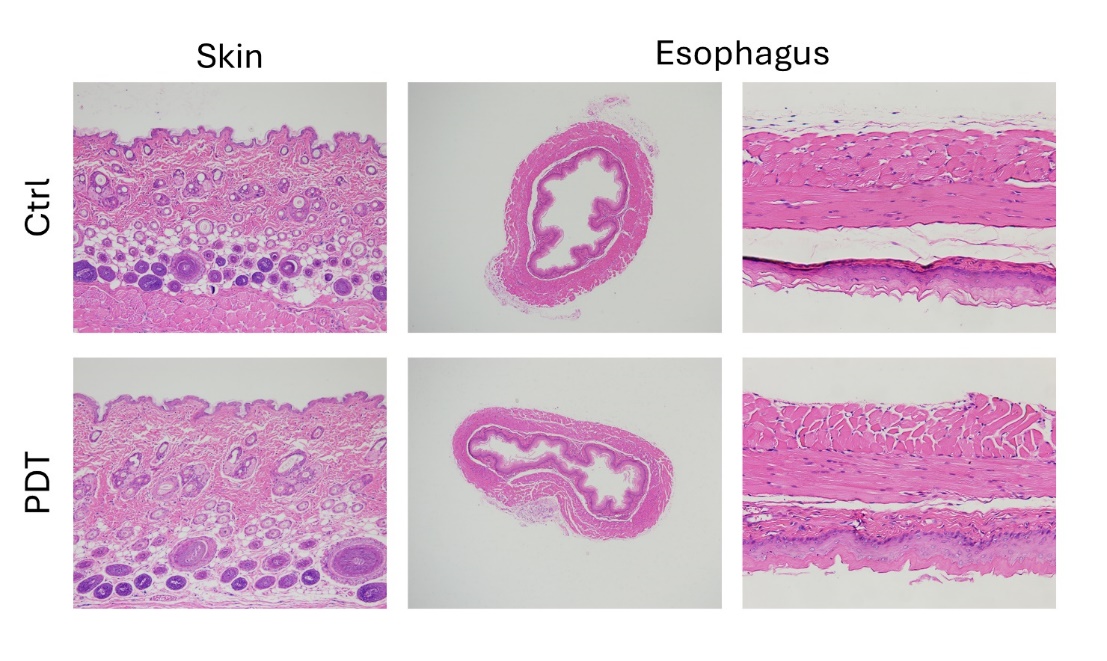


**Figure S14.** Histological examination of skin tissue and oesophagus using H&E staining. Scale bars, 200 μm for the skin and esophagus in longitudinal cutting and 400 μm for the esophagus in crosscut.


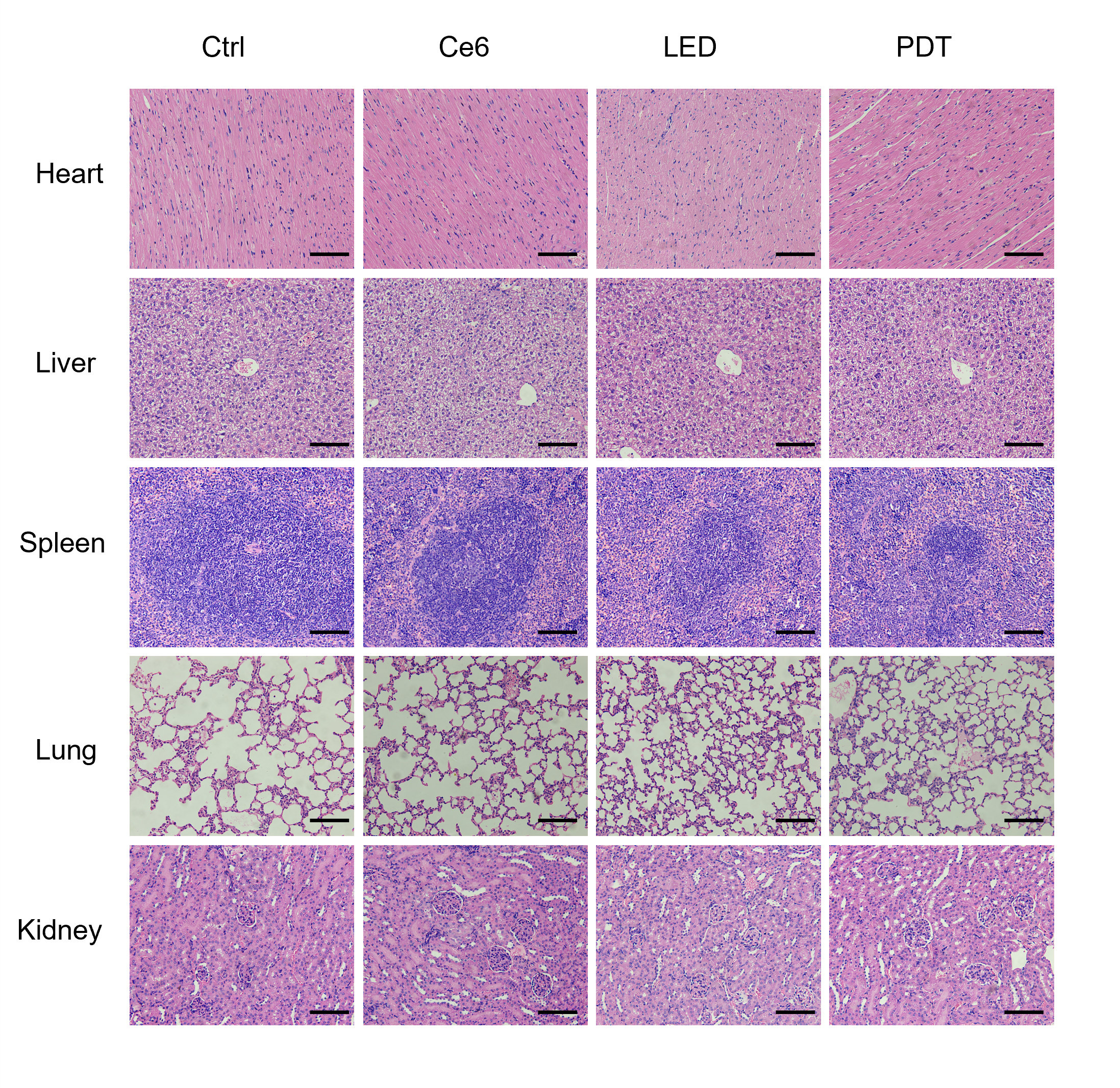


**Figure S15.** Histological morphology of mice major organs after treatment. H&E staining of mice normal organs including heart, liver, spleen, lung, and kidney after various treatments. Scale bars, 100μm.
